# Determination of Potential Lead Compound from *Magnolia officinalis* for Alzheimer’s Disease through Pharmacokinetic Prediction, Molecular Docking, Dynamic Simulation, and Experimental Validation

**DOI:** 10.3390/ijms251910507

**Published:** 2024-09-29

**Authors:** Kumju Youn, Mira Jun

**Affiliations:** 1 Department of Food Science and Nutrition, Dong-A University, Busan 49315, Republic of Korea; kjyoun@dau.ac.kr; 2 Center for Food & Bio Innovation, Dong-A University, Busan 49315, Republic of Korea; 3 Department of Health Sciences, The Graduate School of Dong-A University, Busan 49315, Republic of Korea

**Keywords:** Alzheimer’s disease, Aβ, AChE, BACE1, GSK-3β, honokiol, QC

## Abstract

Amyloid β protein (Aβ) deposition has been implicated as the molecular driver of Alzheimer’s disease (AD) progression. The modulation of the formation of abnormal aggregates and their post-translational modification is strongly suggested as the most effective approach to anti-AD. Beta-site APP-cleaving enzyme 1 (BACE1) acts upstream in amyloidogenic processing to generate Aβ, which rapidly aggregates alone or in combination with acetylcholinesterase (AChE) to form fibrils. Accumulated Aβ promotes BACE1 activation via glycogen synthase kinase-3β (GSK-3β) and is post-translationally modified by glutaminyl cyclase (QC), resulting in increased neurotoxicity. A novel multi-target inhibitor as a potential AD agent was identified using an in silico approach and experimental validation. *Magnolia officinalis*, which showed the best anti-AD activity in our preliminary study, was subjected to analysis, and 82 compounds were studied. Among 23 compounds with drug-likeness, blood–brain barrier penetration, and safety, honokiol emerged as a lead structure for the inhibition of BACE1, AChE, QC, and GSK-3β in docking and molecular dynamics (MD) simulations. Furthermore, honokiol was found to be an excellent multi-target inhibitor of these enzymes with an IC_50_ of 6–90 μM, even when compared to other natural single-target inhibitors. Taken together, the present study is the first to demonstrate that honokiol acts as a multiple enzyme inhibitor with an excellent pharmacokinetic and safety profile which may provide inhibitory effects in broad-range areas including the overproduction, aggregation, and post-translational modification of Aβ. It also provides insight into novel structural features for the design and discovery of multi-target inhibitors for anti-AD.

## 1. Introduction

Alzheimer’s disease (AD) is a complex neurodegenerative disorder characterized by progressive and irreversible memory and cognitive dysfunctions. AD is the leading cause of dementia in the elderly, accounting for 60–80% of cases worldwide. According to the World Health Organization (WHO), the number of people with dementia worldwide is expected to increase sharply from 55 to 139 million by 2050 [1]. AD is caused by both genetic and environmental factors. Presenilin 1, presenilin 2, and the amyloid precursor protein (APP) were found to have a significant impact on early-onset AD, while the APOE-ε4 allele was a genetic risk factor for late-onset AD [2]. Efforts to develop AD treatments have primarily focused on cholinergic neurotransmission, which is mediated by acetylcholine (ACh) and terminated by acetylcholinesterase (AChE) and butyrylcholinesterase (BuChE) activity. It plays an important role in neuronal function, learning, memory, and other aspects of cognition. However, the single cholinesterase inhibitor strategy in AD treatment provides temporary improvements in cognitive function, but does not alter the underlying course of the disease.

The deposition of senile plaques composed of β-amyloid (Aβ) peptides is one of the pathological hallmarks of AD brains. β-Secretase is the first proteolytic enzyme in amyloid processing that initiates the cleavage of the APP to produce an N-terminal fragment and a membrane-bound C-terminal fragment. The C-terminal fragment is then cleaved by γ-secretase to release Aβ, causing oxidative stress, mitochondrial dysfunction, and neuroinflammation [3,4]. Beta-site APP-cleaving enzyme 1 (BACE1) is the most promising β-secretase candidate in the aspartyl protease family and is highly expressed in the central nervous system (CNS). BACE1 is responsible for the rate-limiting step in Aβ production, and abnormally elevated activities and expression levels of this enzyme have been observed in the brains of AD patients [5]. Overproduced Aβ fragments by the enzyme undergo aggregation and fibrillation to form senile plaques, which disrupt cellular communication and trigger inflammatory responses, and ultimately neuronal cell death [6]. Recently, Aβ aggregation has been promoted by AChE through the interaction between the cationic sequence of Aβ and the hydrophobic, peripheral anionic-binding site (PAS) on AChE [7,8]. In addition, there is increasing evidence that the AChE-Aβ complexes are more stable and neurotoxic than Aβ itself [5,9].

Among the different Aβ species, Aβ1-42 is known to be the major component of senile plaques due to its hydrophobic and fibrillogenic potential. However, recent studies have shown that the composition of Aβ in the senile plaques is highly diverse and heterogeneous due to various post-translational modifications of Aβ [10]. In particular, the pyroglutamate-modified Aβ (AβpE3-42) represents an important variant of the total Aβ species in AD brains. Compelling evidence has shown that Aβ pE3-42 has a higher aggregation capacity and acts as a seed that promotes Aβ assembly, resulting in a denser, more stable, and cytotoxic species than that of full-length Aβ1-42 [11]. Of particular interest is the glutaminyl cyclase (QC), which plays an important role in the conversion and formation of the AβpE3-42. QC is highly expressed in the human brain and is upregulated in AD, leading to an increase in AβpE3-42 formation [12].

Interestingly, recent studies have shown that Aβ accumulation in the AD brain is closely linked to the production of hyperphosphorylated tau protein, which is one of the pathological hallmarks of AD [13,14]. Aβ promotes tau hyperphosphorylation by activating tau kinases. As one of the major tau kinases, glycogen synthase kinase-3β (GSK-3β) is highly expressed in the central nervous system (CNS) and is found hyperactive in the brain of AD patients [15]. To induce tau hyperphosphorylation, Aβ can interfere with the normal Wnt signaling pathway, which blocks Wnt-mediated GSK3-3β inactivation, or it can directly activate GSK3-3β [16]. Furthermore, the evidence suggests that overactivated GSK3-3β upregulates the activation and expression of BACE1 [17,18].

Natural products with diverse chemical structures offer the opportunity to discover new lead compounds that can be converted into drugs, owing to their broad spectrum of biological activities, diversity, and safety. After screening 20 herb and plant extracts for novel inhibitors of Aβ aggregation, *Magnolia officinalis* (*M. officinalis*) with the most potent effect was selected to demonstrate novel multi-target candidates for AD prevention. *M. officinalis* is a traditional herb used in China, Japan, and Korea as tea and medicine for the treatment of gastrointestinal disorders, diabetes, depression, nervousness, and anxiety [19]. Neolignans such as honokiol (17–96.5 mg/g) and magnolol (21.3–91.9 mg/g) have been shown to be the main constituents of *M. officinalis* [20,21]. According to the previous studies, *M. officinalis* extract improved the neurotrophic effects and prevented cognitive impairment caused by various factors, including scopolamine, inflammation, and Aβ [22,23]. The neuroprotective effects of honokiol and magnolol have been reported to demonstrate targeting memory impairment, Aβ toxicity, oxidative stress, neuroinflammation, mitochondrial dysfunction, cholinergic deficit, intestinal flora, and a neurotrophic effect [24,25,26]. To the best our knowledge, it is the first attempt to design the discovery of multi-target inhibitors against BACE1, AChE, QC, and GSK3-3β in *M. officinalis*-derived compounds as the preventive and/or therapeutic strategy for AD.

**Figure 1:**
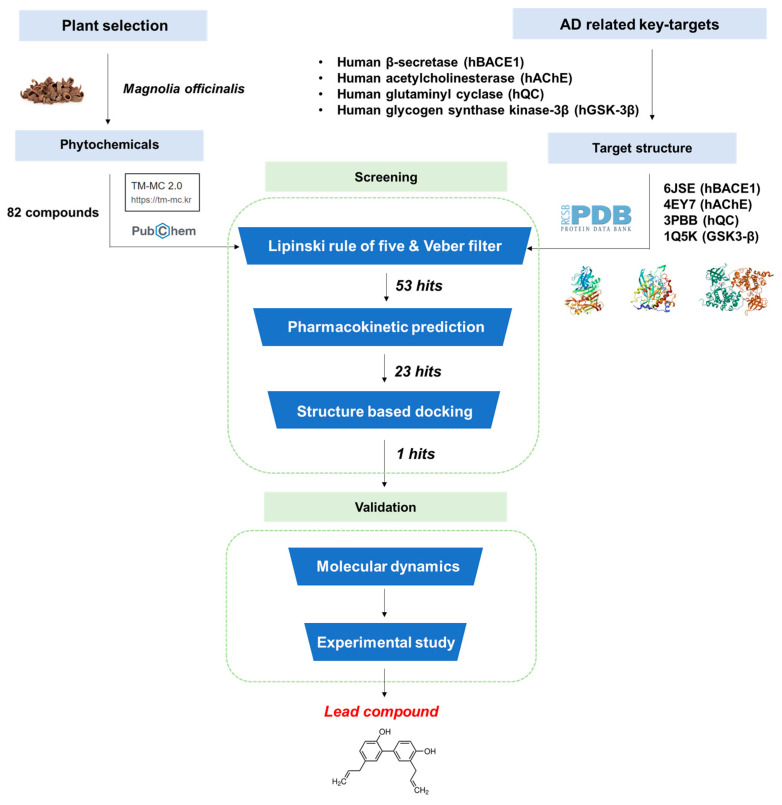
Workflow of the study.

In the present study, pharmacokinetic prediction using in silico absorption, distribution, metabolism, and excretion (ADME) was performed to identify compounds with drug-like properties and blood–brain barrier (BBB) penetration efficiency. Furthermore, computational docking and molecular dynamics (MD) simulations were used to analyze whether the compound could reach the target enzyme to produce a biological effect and interact with target sites. Finally, in vitro biological studies allowed us to validate novel multifunctional candidates with activity against human BACE1 (hBACE1), hAChE, hQC, and hGSK-3β. A schematic of the workflow used in this study is shown in Figure 1.

## 2. Results

### 2.1. Drug-Like Properties of the Compounds in M. officinalis

Based on compound mining from the TM-MC 2.0, 82 compounds in *M. officinalis* reported in the qualitative chromatographic analysis literature were collected without duplicates, and their chemical structures are shown in Figure S1. TM-MC 2.0 is the largest database of chemical compounds from the medicinal materials listed in northeast Asian pharmacopeias [27]. These compounds were selected to predict drug-like properties based on their physicochemical indicators using the Lipinski and Veber rule. The first rule states that a compound has poor absorption and permeation when the following criteria are met: molecular weight >500 Da, lipophilicity (LogP) and hydrogen bond donors >5, and hydrogen bond acceptors >10. The second is an extension of Lipinski’s rule, which states that the bioavailability of a compound further increases when there are fewer than 10 rotational bonds and the polar surface area is not greater than 140. A total of 53 compounds were obtained that did not violate these rules and were analyzed for further pharmacokinetic predictions (Table 1).

### 2.2. Pharmacokinetic Properties of Compounds in M. officinalis

The BBB tightly controls the influx and efflux of substances in order to maintain homeostasis and proper neuronal function. In addition, P-glycoprotein (P-gp) is the major efflux transporter of the BBB, responsible for transporting its substrates from the brain endothelial cells into the blood [28]. A selection of 53 compounds were analyzed for their ability to cross the BBB and act as P-gp substrates. BBB penetration was evaluated using the lipophilicity and topological polar surface area of the compounds. As a result, 46 compounds were able to cross the BBB, but only 24 of them did not act as p-gp substrates, indicating that 24 candidates could reach their target enzymes in vivo.

To predict the toxicity risk of our hit compounds, the AMES activity, maximum tolerated dose in humans (MTD), oral rat acute toxicity (LD_50_), and chronic oral toxicity in rats were evaluated (Table 2). Except for compound (42), 23 compounds tested negative in the AMES test, indicating no toxicity in terms of the mutagenic potential. The MTD is the toxic dose threshold for molecules in Phase I clinical trials. Based on the default value of the MTD (0.477 log mg/kg/day), a dose less than or equal to the baseline was considered low, whereas a dose greater than the baseline was considered high. Compounds (19), (20), (26), (38), (42), (43), (62), (67), and (78) with >0.477 log(mg/kg/day) had high MTD values, whereas the remaining 15 compounds had a low one. In addition, the predicted values of LD_50_ and chronic oral rat toxicity confirmed that the 23 compounds had low toxicity. Therefore, together with their ADME properties, the 23 identified compounds showed desirable pharmacokinetic properties as potential anti-AD candidates.

### 2.3. Molecular Docking on Enzymatic Targets: hBACE1, hAChE, hQC, and hGSK-3ß

The binding free energy level between the ligand and receptor reflects the binding potency, with lower energy levels indicating stronger binding. In general, the binding activity is considered strong when the binding energy is less than −7.0 kcal/mol [29]. However, in the present study, the cut-off for the binding energy was set at ≤−7.5 kcal/mol. As illustrated in Table 3, 14 compounds, except for 9, had potent binding interactions with at least one AD-related target. However, only one hit (honokiol) was found to have high binding potency for all targets, and this was selected as the lead compound. The docking results were evaluated by the re-docking of all target enzymes with their native ligands to validate the process accuracy.

### 2.4. Binding Interaction of the Best Compound with Multi-Enzyme Targets

**Figure 2:**
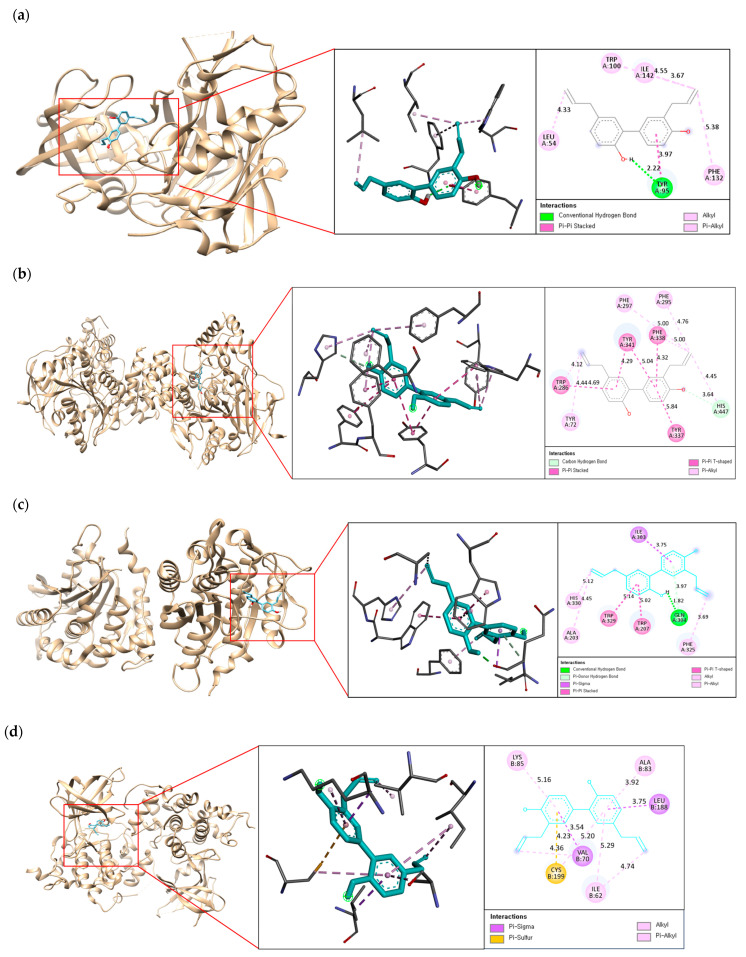
The best docking poses of honokiol with (**a**) hBACE1; (**b**) hAChE(B); (**c**) hQC; and (**d**) GSK-3β.

A post-docking simulation between the lead compound and AD-related targets was performed to further identify the binding mode, binding site residues, and interaction type. The active site of BACE1 contains a catalytic ASP dyad formed by two aspartate residues such as ASP 32 and ASP228. Furthermore, LYS 9-TYR14 (10s loop), VAL 67-GLU 77 (flap), GLY 158-LEU 167 (insert A), TRP 270-THR 274 (insert D), and ASP 311-ASP 317 (insert F) are known to facilitate the substrate’s entry and binding to the active site through their movements [30]. As shown in Figure 2a, honokiol exhibited hydrogen bond interactions with TYR 95 and hydrophobic alkyl interactions with LEU 54, TRP100, PHE 132, and ILE 142. Interestingly, the compound did not directly interact with the catalytic aspartate dyad or the critical region that allows substrate binding to the enzyme, demonstrating that the compound is a noncompetitive inhibitor docked at the noncatalytic site of BACE1.

The PAS of hAChE involved in Aβ aggregation is located at the entrance of the active site gorge and consists of residues such as TYR 72, TYR 124, ASP 74, TYR 341, and TRP 286 [31]. Figure 2b showed that honokiol formed one carbon–hydrogen bond with HIS 447; four π-π interactions with TRP 286, TYR 337, PHE 338, and TYR 341; and three π-alkyl interactions with TYR 72, PHE 295, and PHE 297. Honokiol was stably bound to the PAS site of hAChE, suggesting that the compound may prevent Aβ aggregation by blocking the Aβ-hAChE interaction.

The honokiol-hQC complex formed one hydrogen bond with residue GLN 304, two π-stacks with TRP 207 and TRP 329, and three alkyl interactions with residues ALA 203, PHE 320, and HIS330, as shown in Figure 2c. In particular, TRP207, ILE303, TRP329, and PHE 325, involved in hydrophobic interactions, are active-site residues of hQC, suggesting that this compound is a competitive inhibitor of hQC [32].

In the case of honokiol-hGSK-3β, hydrophobic interactions were predominantly formed, as shown in Figure 2d. The positions of VAL 70 and LEU 188 acquired two π–sigma interactions; the CYS 199 residue formed a π–sulfur; while ILE 62, ALA 83, and LYS 85 formed hydrophobic π–alkyl interactions. The complex of honokiol with hGSK-3β provided insight into the binding mode in the allosteric hydrophobic pocket of the enzyme containing ILE 62, VAL 70, and GLY 83 [33].

### 2.5. MD Simulations of Honokiol with hBACE1, hAChE, hQC, and hGSK-3ß

MD simulations predict biological systems at the atomic level using physics-based force fields and reveal the behavior of the protein–ligand complex in a solvated environment, thus complementing the simplified treatment of static protein–ligand binding in molecular docking models. Moreover, simulations allow the study of dynamic interactions such as motions, fluctuations of residues, movement in specific domains, and the dissociation of protein–ligand complexes. In particular, the most commonly used measures in MD simulations are the root mean square deviation (RMSD) and root mean square fluctuations (RMSF). RMSD measures the average distance generated by the displacement of selected atoms for a given time frame with respect to a reference time, which is useful for analyzing the time-dependent motions of a structure. RMSF determined the residue-wise fluctuations of amino acid residues to identify conformational changes of the protein in the complex with the ligand [34]. This validation criterion highlights the importance of specific protein residues in these structural shifts.

**Figure 3:**
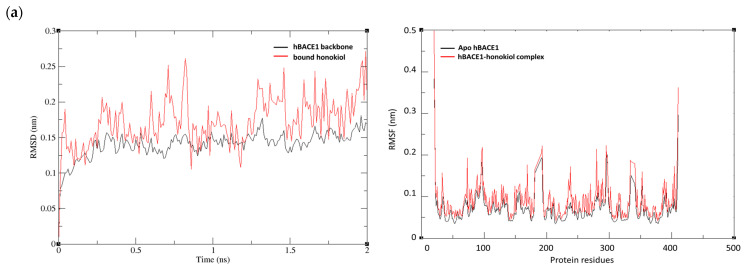

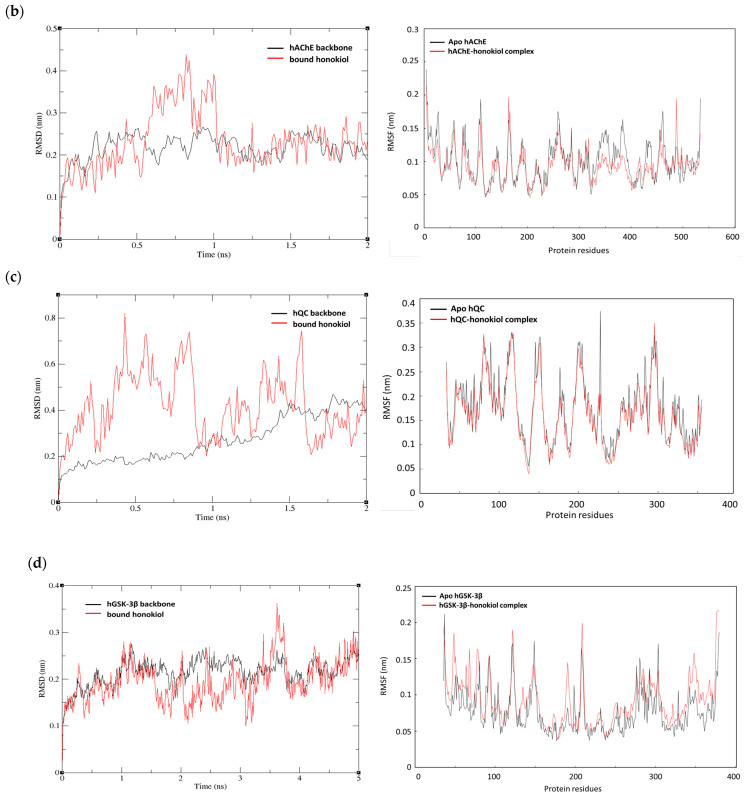
Root mean square deviation (RMSD) and Root mean square fluctuations (RMSF) during MD simulation of (**a**) hBACE1-honokiol complex; (**b**) hAChE-honokiol complex; (**c**) hQC-honokiol complex; and (**d**) GSK-3β-honokiol complex.

The complex of honokiol and four target enzymes with the most negative binding energy was nominated for MD analysis. RMSD was plotted using complete trajectories to analyze the stability of honokiol and its target enzymes. Among the four complexes, the hBACE1-honokiol, the hAChE-honokiol, and the hGSK-3β-honokiol complexes reached equilibrium and fluctuated around their mean values after about 1ns, indicating that these systems were well-behaved thereafter (Figure 3a,b,d). As shown in Figure 3c, the hQC-honokiol complex showed various fluctuations in its RMSD values at 0.5–1.5 to 0.8 nm and then the complex became stabilized at an RMSD value of 0.2–0.5 nm until the end of the MD simulation. Significant changes in RMSD could be caused by various factors such as structural changes or transitions between different states. Although longer simulations may be required to capture exact structural changes or to ensure convergence, the hQC-honokiol complex has reached a structural equilibrium.

For understanding the residues that participated in the causative factors for fluctuations, the RMSF values were plotted. The hBACE1-honokiol complex showed small fluctuations with RMSF values <0.2 nm for key amino acids such as 54, 95, 100, 132, and 142 (Figure 3a). The RMSF result of the hAChE-honokiol complex revealed that four fluctuations occurred at 72, 297, 295, and 447 amino acid residues, which was consistent with the results of the docking simulation (Figure 3b). In the RMSF of the hQC-honokiol complex compared to the apo-protein, fluctuations were observed in the active site regions such as 207, 303, 329, and 325, indicating that the ligand was able to adapt well in the binding pocket of the protein (Figure 3c). The hGSK-3β-honokiol complex also showed fluctuations centered around the amino acids involved in the binding (62, 70, 83, 85, 188, and 189), but with lower RMSF values compared to the other complexes (Figure 3d).

### 2.6. Inhibitory Effects of Honokiol on hBACE1, AChE, hQC, and hGSK-3β

As shown in Table 4, honokiol had a significant effect on hGSK-3β with an IC_50_ value of 6.70 ± 0.03 µM, which surpassed that of rutin, a well-known hGSK-3β inhibitor. This value is still potent when compared to the IC_50_ value of other flavonoid-based natural GSK-3β inhibitors (IC_50_, 7.0–135.5) such as rosmarinic acid, apocynin, psoralidin, savinolic acid A-C, and others [35,36,37]. The compound was also a potent inhibitor of BACE1 (IC_50_, 20.64 ± 0.80 μM) and showed similar effects to the positive control, quercetin. In comparison to other known natural compounds (IC_50_ value ranging from 22.5 to 63 μM), including quercetin, myricetin, genistein, and in flavonoids, epiberberine and groenlandicine in alkaloids, 2,2′,4′-trihydroxychalcone acid and cardamonin in chalcones, and gracilin L and ginsenoside Rg1 in terpenes, honokiol was found to have good inhibitory potential against BACE1 [38,39,40]. The inhibitory activities of honokiol with an IC_50_ range of 78–90 µM against AChE and hQC were relatively moderate compared to its potent effects on GSK-3β and BACE1, but still significant, considering the wide IC_50_ ranges of natural AChE inhibitors (from 0.63 µM to 226 µM), such as alkaloids, flavonoids, stilbenes, terpenoids, and glycosides, and natural QC inhibitors (from 16 to 250 µM), including sulforipids from microalgae, oleuropein aglycones from olive oil, and apigenin derivatives [41,42,43,44].

### 2.7. Kinetic Studies of hBACE1, AChE, hQC, and hGSK-3β Inhibition by Honokiol

**Figure 4:**
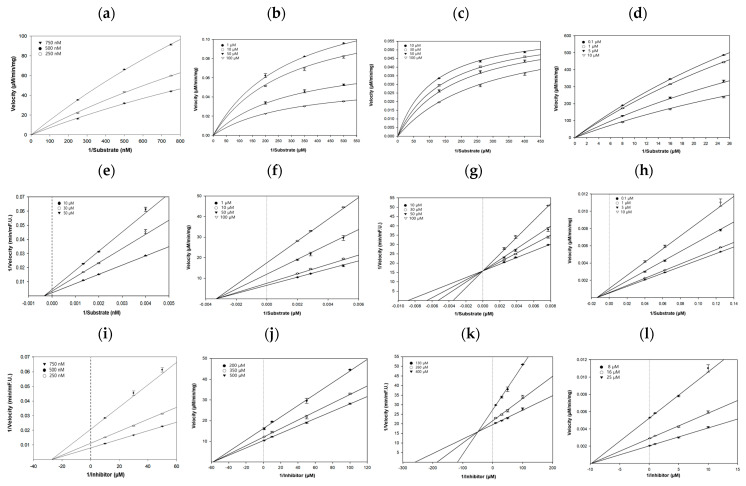
Michaelis–Menten, Dixon, and Lineweaver–Burk plots for the inhibitory properties of honokiol against hBACE1 (**a**,**e**,**i**), hAChE (**b**,**f**,**j**), hQC (**c**,**g**,**k**), and hGSK-3β (**d**,**h**,**l**). Michaelis–Menten and Lineweaver–Burk plots were analyzed in the presence of different inhibitor concentrations and Dixon plots showed the effects of the presence of different substrate concentrations.

To gain insight into inhibition mode and kinetic parameters, the Michaelis–Menten curves, Dixon plots, and Lineweaver–Burk plots were used. As shown in Figure 4a,e,i, the inhibition constant (Ki value) was 27.3 μM, which was determined from the negative *x*-axis at the intersection point of three lines in the Dixon plot. In addition, the Lineweaver–Burk plot showed that the maximum reaction velocity (Vmax) decreased and the Michaelis–Menten constant (Km) remained unchanged with increasing inhibitor concentrations, indicating that honokiol is a noncompetitive BACE1 inhibitor that does not involve the catalytic dyad, consistent with the docking results. As BACE1 is a membrane-bound aspartyl protease with a catalytic Asp dyad responsible for cleaving APP through an acid–base mechanism, direct inhibition via these aspartyl residues may have detrimental effects [45].

For AChE inhibition by honokiol, the non-competitive inhibition mode was well-fitted, and its Ki value from the Dixon plot was 58.2 μM, as shown in Figure 4b,f,j. Both the enzyme kinetic and docking simulation results revealed the strong binding of honokiol bound to the PAS of AChE, which may limit the Aβ aggregation via the conformational change of Aβ to the β-sheet fold by AChE and reduce the resulting neuronal damage.

As shown in Figure 4c,g,k, the Ki value of honokiol obtained from the Dixon plot was 49.4 μM. In the Lineweaver–Burk plot, the Km value increased in the presence of different concentrations of honokiol, while Vmax remained unchanged as the intercept on the *y*-axis, suggesting that our compound was a competitive inhibitor of hQC. This result was consistent with the docking analysis, showing a close interaction with the active region of QC, which consists of a hydrophobic entrance, a narrow binding pocket, and a catalytic zinc ion located at the bottom of the pocket.

The Lineweaver–Burk plot for hGSK-3β inhibition by honokiol was well fitted to the non-competitive inhibition mode, and its Ki value from the Dixon plot was 9.8 μM, as shown in Figure 4d,h,l. It is indicated that honokiol binds somewhere other than the active sites of hGSK-3β, changing the overall shape of the site for substrates so that it does not fit as well as before, slowing or preventing the reaction with the substrate.

## 3. Discussion

In the present study, potential candidates from *M. officinalis* were screened using an in silico multitarget prediction system to identify drug-likeness, BBB permeability, and toxicity. Honokiol is a proven multiple enzyme inhibitor whose binding affinity and conformation can be predicted. Interestingly, honokiol had a very similar mode of interaction to the FDA-approved drug donepezil, sharing the same hydrophobic binding with TRP 286, PHE 295, PHE 338, and TYR 341 in the PAS site of hAChE [46]. However, in binding to BACE1, honokiol exhibited strong binding potential comparable to that of donepezil, but with different binding sites [47]. Donepezil is a competitive inhibitor of BACE1, binding to the ASP dyad (ASP32 and ASP228) in the active site, whereas honokiol was shown to be a non-competitive BACE1 inhibitor, binding to the non-catalytic sites such as LEU 54, TYR95, TRP100, PHE 132, and ILE 142. In addition, to the best of our knowledge, there are yet no studies on the interaction between donepezil and hQC or hGSK-3β. From the MD simulation studies, it was found that the docked complexes between hBACE1, hAChE, hQC, and hGSK-3β and honokiol were stable and the maximum RMSD was found to be less than 3 Å. Importantly, our study combined biological experiments to directly validate the biological activity of the selected lead compound (honokiol), and the compound was identified as the novel multi-target-directed AD candidate with inhibitory activities against four targets. Moreover, the binding mechanism of the lead compound with key AD-related enzymes was elucidated, providing valuable information for the subsequent design of natural anti-AD candidates based on the mechanism of action.

AD is a progressive neurodegenerative disorder for which there is currently no effective treatment. Multi-target inhibitors are more effective than the conventional single-target inhibitors due to their cumulative efficacy against individual targets, making them a suitable strategy for multifactorial diseases such as AD. It is not only important that the multi-target inhibitors have balanced activities against the targets of interest, but also that the pharmacokinetic properties are optimized for oral administration and permeation across the BBB. Indeed, the unfavorable pharmacokinetic properties of the reported multi-target inhibitors remain to be the main barrier to the further clinical translation of these compounds. The present study has demonstrated that honokiol has stringent physicochemical properties for effective BBB penetration to exert its anti-AD activity. In addition, Serrica et al. showed that no side effects have been reported in humans associated with the use of honokiol or herbal formulations containing the compound [21]. However, honokiol may have the potential toxicity to interact with off-targets that would otherwise cause side effects. Therefore, it is necessary to further confirm that the compound has sufficient selectivity against off-targets.

Honokiol (3,5′-diallyl-4,2′-dihydroxybiphenyl) and magnolol (5,5′-diallyl-2,2′-dihydroxybiphenyl), the major constituents of *M. officinalis*, are neolignane isomers composed of two phenylpropanoid units linked by aromatic C-C bonds, but in the present study, their pharmacological effects appeared to differ greatly. In our preliminary study, honokiol and magnolol were evaluated for anti-AD activity. It was found that magnolol had no significant inhibitory effect on hBACE1, hAChE, hQC, and hGSK-3β (IC_50_ >100 μM) and lower anti-Aβ_1-42_ aggregation effects than that of honokiol. Previous studies have shown that honokiol was more potent than magnolol on antioxidant and neurotrophic activities, similar to our present findings [19,48,49]. This suggested that the potent multi-enzyme inhibitory effect of honokiol was probably related to the difference in the phenolic function induced by their molecular substituents. Honokiol contains two hydroxyl groups at *ortho*- and *para*-positions, whereas magnolol contains two hydroxyl groups both at *ortho*-positions. Interestingly, our result demonstrated that the 4′-hydroxyl and the 5-aryll groups of honokiol are responsible for its potential activity, whereas the intramolecular hydrogen bond formed between di-ortho-hydroxyl groups of magnolol appears to be associated with its lower activity.

Previous studies have provided the neuroprotective effects of honokiol owing to its potent antioxidant activity and anti-excitotoxicity, which are mainly related to a glutamate receptor blockade and the reduction of neuroinflammation [50,51,52]. In addition, a growing body of evidence has demonstrated the significant protective effects of honokiol against cognitive impairment induced by various factors such as aging, scopolamine, chronic restraint stress, and synaptic damage [53,54,55,56]. Accumulating evidence shows that the metal ions were dysregulated in the vulnerable brain regions of AD patients, which was highly associated with Aβ deposition, tau hyperphosphorylation, neuronal loss, as well as neuroinflammation [57]. In particular, iron accumulation triggers ROS production and lipid peroxidation, leading to ferroptosis [58]. To date, the interaction of honokiol with metal ions has not been studied. However, given the multi-purpose effects of polyphenols, honokiol may have an inhibitory effect on ferroptosis through direct interactions with metal ions.

According to the suppressive role of honokiol on Aβ toxicity, the compound had the ability to enhance neuronal survival and attenuate the apoptosis of the hippocampal neuron in an Aβ oligomer-induced mouse model [59]. Furthermore, the neuroprotection of honokiol against Aβ-induced damage was demonstrated in a transgenic *Caenorhabditis elegans* model, an in vivo system containing human Aβ_1-42_ [60]. However, to the best of our knowledge, no direct multiple effects of honokiol on BACE1, GSK-3β, AChE, and QC activities have been reported. From the next study, the interaction networks of target enzymes will be conducted to justify the selection of target enzymes and to elucidate the molecular mechanisms in AD prevention.

## 4. Materials and Methods

### 4.1. Collection of M. officinalis Compounds

The compounds of *M. officinalis* were collected from the TM-MC 2.0 database (Seoul, Republic of Korea; https://tm-mc.kr/search.do; last update: 25 September 2023). Of the 122 compounds, a total of 82 compounds were selected after eliminating duplicate structures. For in silico pharmacokinetic prediction, structural information on the compounds, such as data regarding the SMILES and CID, was collected from the PubChem database (Bethesda, MD, USA; https://pubchem.ncbi.nlm.nih.gov/; last update: 30 September 2023).

### 4.2. Drug-Likeness and ADMET Analysis

Chemical candidates were screened using the SwissADME database (accessed on 25 November 2023) based on the physicochemical properties of the drug-likeness index. Drug-likeness candidature was implemented by Lipinski’s Rule of Five and Veber’s Rule based on the physicochemical properties of the tested compounds, such as molecular weight, hydrogen bond donor or acceptor, lipophilicity (LogP), molecular refractivity, number of atoms, and polar surface area. The pharmacokinetic properties of the selected compounds, including both p-glycoprotein substrate and BBB permeability, were calculated using SwissADME (Lausanne, Swaziland; http://www.swissadme.ch/) and pkCSM (Melbourne, Australia; https://biosig.lab.uq.edu.au/pkcsm/).

### 4.3. Molecular Docking Simulation

The X-ray crystallographic structures of key enzyme targets, including hBACE1 (PDB ID: 6JSE), hAChE (PDB ID: 4EY7), hQC (PDB ID: 3PBB), and hGSK-3β (PDB ID: 1Q5K), were obtained from the RCSB Protein Data Bank (PDB) based on origin species and ligand similarity criteria. Before molecular docking, all co-crystallized ligands and water molecules of the targets were removed using UCSF Chimera (San Francisco, CA, USA; https://www.cgl.ucsf.edu/chimera/). Autodock 1.1.2 software (San Diego, CA, USA) was used to dock and search for the optimal conformation, which was determined by the binding mode and its corresponding binding affinity. The types of interactions between the compounds and targets were analyzed using Maestro (Schrödinger, New York, NY, USA; version 13.8) and the Biovia Discovery Studio Visualizer (Dassault Systèmes, Boston, MA, USA; https://www.3ds.com/products/biovia/discovery-studio/visualization). The compound with the highest binding affinity for all AD targets was selected for MD and experimental validation.

### 4.4. MD Simulation

To measure the interaction stability of the hit compound AD targets, the best poses obtained from the post-docking studies were evaluated using 2–5-ns MD simulations. All MD simulations were performed using GROMACS version 2023.3 in a Linux environment. Protein topologies were generated using the CHARMM 27 force field with the TIP3P water model; the honokiol topology was generated using the AMBER force field. The complex was solvated with single-point charge water, placed on a dodecahedron box, and neutralized by adding Na+ and Cl− at a salt concentration of 0.15 M. Energy minimization was performed to relax the entire system using the steepest descent integrator for 50,000 steps. After equilibration, a total of 2–5 ns of simulation was performed for each system while maintaining the temperature at 300 K.

### 4.5. Reagents

Honokiol (≥98% purity), quercetin (≥95% purity), galantamine, resveratrol (≥99% purity), rutin (≥98% purity), AChE, 5,5′-dithio-bis-[2-nitrobenzoic acid] (DTNB), DMSO, HEPES, and acetylthiocholine iodide (ACTI) were purchased from Sigma-Aldrich (St. Louis, MO, USA). The BACE1 fluorescence resonance energy transfer (FRET) assay kit was obtained from Invitrogen (Pan Vera, Madison, WI, USA). hQC, H-Gln-AMC hydrobromide, and pyroglutamyl peptidase (pGAPase) were purchased from R&D Systems (Minneapolis, MN, USA). Active hGSK-3β, substrate (YRRAAVPPSPSLSRHSSPHQ (pS) EDEEE), buffer (Tris-HCl, MgCl2, and BSA, pH 7.4), and ADP-Glo™ kinase assay kit were purchased from Promega (Madison, WI, USA).

### 4.6. hBACE1 Inhibitory Activity

The FRET-based assay was used to evaluate the anti-hBACE1 activity and was performed according to the manufacturer’s instructions. The fluorescence intensity (excitation, 545 nm; emission, 590 nm) was measured using a Synergy H1 microplate reader (BioTek Instruments, Winooski, VT, USA). The percentage BACE1 inhibitory activity was calculated using the following formula:

Inhibition (%) = [1 − (S − S0)/(C − C0)] × 100(1)

where C is the control fluorescence without the sample after 60 min of incubation, C0 is the control fluorescence at time 0, S is the test sample fluorescence after 60 min of incubation, and S0 is the test sample fluorescence at time 0. The concentrations of honokiol used for IC_50_ value were 10, 30, 50, and 100 μM.

### 4.7. AChE Inhibitory Activity

The AChE inhibitory activity was measured spectrophotometrically using the modified Ellman method [46]. First, AChE (0.8 U/mL) was added to a mixture containing sodium phosphate buffer (0.1 M; pH 8.0), DTNB (5 mM), and a test sample, followed by incubation at 37 °C for 15 min in 96-well microplates. Next, ACTI (500 µM; substrate for AChE) was added to the mixture to activate the reaction. The enzymatic hydrolysis of ACTI was monitored using a Synergy H1 microplate reader to detect the formation of a yellow anion (5-thio-2-nitrobenzoic acid) resulting from the reaction between DTNB and thioiodide released by the enzyme at 405 nm. Four different concentrations of honokiol (ranging from 1 to 100 μM) were tested for IC_50_ value.

### 4.8. hQC Inhibitory Activity

The inhibitory activity of hQC was evaluated using H-Gln-AMC hydrobromide (400 μM) as the substrate in a fluorometric assay. The buffer consisted of 25 mM HEPES (pH 7.0; adjusted with HCl). The hQC (10 μg/mL) and its auxiliary enzyme pGAPase (50 units) were pre-diluted 1:250 in HEPES. A typical reaction mixture consisted of the substrate, test compounds, and pGAPase. After incubation in black 96-well plates for 10 min at 37 °C, the reaction was initiated by adding hQC solution. The fluorescence intensity of the samples was measured at excitation/emission wavelengths of 380/460 nm, which correlated with the amount of AMC, the end product of the enzymatic process. For the IC_50_ values, honokiol concentrations of 10, 30, 50, and 100 μM were used.

### 4.9. hGSK-3β Activity

Inhibition of hGSK-3β activity was evaluated using the Kinase-Glo assay. To initiate the reaction, test compound and hGSK-3β (20 ng) were added to each well of a 384-well black plate, followed by the addition of 20 μL of assay buffer containing GSM (hGSK-3β substrate peptide, 25 μM) and ATP (final concentration, 1 μM). The final DMSO concentration in the reaction mixture did not exceed 1%. The reaction mixture was then incubated at 37 °C for 30 min, followed by the addition of the ADP-GloTM reagent (20 μL) to terminate the enzymatic reaction and deplete the remaining ATP. The kinase detection reagent was added to the well plate and glow-type luminescence was recorded using a Synergy H1 microplate reader. Honokiol concentrations of 0.1, 1, 5, and 10 μM were used to determine IC_50_ values.

### 4.10. Enzymatic Kinetics on hBACE1, hAChE, hQC, and hGSK-3β

Dixon plots were obtained in the presence of different concentrations of substrates for AD-related target enzymes. Michaelis–Menten and Lineweaver–Burk plots were constructed in the absence or presence of various concentrations of honokiol. The Enzyme Kinetic™ module of SigmaPlot™ version 12.3 (Systat Software, Inc., San Jose, CA, USA) was used to calculate kinetic parameters, such as the Ki, Vmax, and Km.

### 4.11. Statistical Analysis

All the results are presented as the means ± standard deviations (SDs) of three independent experiments. Statistical significance was analyzed using SAS (version 9.3; Cary, NC, USA) and Duncan’s multiple-range test.

## 5. Conclusions

The lack of an effective treatment for AD creates the need for candidates that simultaneously regulate the overproduction, aggregation, and post-translational modification of Aβ and the hyperphosphorylation of tau, the processes underlying the disease. Therefore, this study focused on the discovery of novel multifunctional ligands for the prevention of AD. Honokiol was shown for the first time to be a potent multi-target inhibitor of hBACE1, hAChE, hQC, and hGSK-3β through the efficient integration of in silico approaches and biological experiments. Although further in vivo studies are required, this compound has specific advantages over previously reported single and/or dual inhibitors owing to its unique inhibitory mechanism, and the results of pharmacokinetic predictions further support its applicability as a multifunctional anti-AD lead compound.

## Figures and Tables

**Table 1:** In silico pharmacophore modeling of selected 53 compounds in *M. officinalis*.

Compound	Drug-Likeness Property		Pharmacokinetic Profiles	
	Lipinski’s Rule	Veber’s Rule	BBB Penetration	P-gp Substrate
(+)-Syringaresinol (1)	Yes	Yes	No	Yes
Khusilol (2)	Yes	Yes	Yes	No
Asimilobine (3)	Yes	Yes	Yes	Yes
Magnoflorine (4)	Yes	Yes	Yes	Yes
Tembetarine (5)	Yes	Yes	Yes	Yes
Ferulic acid methyl ester (6)	Yes	Yes	Yes	No
3-Pinanone (9)	Yes	Yes	Yes	No
6,6-Dimethyl-2-methylene-bicyclo[2.2.1]-heptan-3-one (11)	Yes	Yes	Yes	Yes
Xanthoplanine (13)	Yes	Yes	Yes	Yes
N-Methylbulbocapnine (14)	Yes	Yes	Yes	Yes
N-Methylcoclaurine (15)	Yes	Yes	Yes	Yes
N-Nornuciferine (16)	Yes	Yes	Yes	Yes
Anonaine (18)	Yes	Yes	Yes	Yes
Borneol (19)	Yes	Yes	Yes	No
Borneol acetate (20)	Yes	Yes	Yes	No
Caffeic acid methyl ester (21)	Yes	Yes	Yes	No
Camphor (23)	Yes	Yes	Yes	No
Caryophyllene oxide (25)	Yes	Yes	Yes	No
cis-Linalool oxide (26)	Yes	Yes	Yes	No
Corytuberine (28)	Yes	Yes	Yes	Yes
Dienestrol (29)	Yes	Yes	Yes	No
Esculetin (32)	Yes	Yes	No	No
Gallic acid (34)	Yes	Yes	No	No
Glaucine (36)	Yes	Yes	Yes	Yes
Hexanal (38)	Yes	Yes	Yes	No
Honokiol (39)	Yes	Yes	Yes	No
Isomagnolol (42)	Yes	Yes	Yes	No
Limonene (43)	Yes	Yes	Yes	No
Lirinidine (44)	Yes	Yes	Yes	Yes
Liriodenine (45)	Yes	Yes	Yes	Yes
Lotusine (46)	Yes	Yes	Yes	Yes
Magnaldehyde D (47)	Yes	Yes	Yes	No
Magnocurarine (48)	Yes	Yes	Yes	Yes
Magnolignan A (49)	Yes	Yes	No	No
Magnolignan C (50)	Yes	Yes	No	No
Magnolol (51)	Yes	Yes	Yes	No
Michelalbine (56)	Yes	Yes	Yes	Yes
Nandigerine (58)	Yes	Yes	Yes	Yes
Nornuciferine (61)	Yes	Yes	Yes	Yes
Obovatol (62)	Yes	Yes	Yes	No
Palmidin B (65)	Yes	Yes	No	No
Phenol (67)	Yes	Yes	Yes	No
Puterine (69)	Yes	Yes	Yes	Yes
Randaiol (70)	Yes	Yes	Yes	No
Reticuline (71)	Yes	Yes	Yes	Yes
Roemerine (72)	Yes	Yes	Yes	Yes
Syringin (73)	Yes	Yes	No	No
Xanthoplanine (74)	Yes	Yes	Yes	Yes
α-Eudesmol (76)	Yes	Yes	Yes	No
α-Terpineol (78)	Yes	Yes	Yes	No
β-Eudesmol (79)	Yes	Yes	Yes	No
γ-Eudesmol (80)	Yes	Yes	Yes	No
γ-Gurjunene epoxide (81)	Yes	Yes	Yes	No

**Table 2:** In silico toxicity profiles of screened 24 compounds using pkCSM.

Ligand	AMES Toxicity	Maximum Tolerated Dose in Human	Oral Rat Acute Toxicity (LD_50_, mol/kg)	Oral Rat Chronic Toxicity (log mg/kg)
Khusilol (2)	No	−0.279	1.673	1.335
Ferulic acid methyl ester (6)	No	0.309	1.653	1.908
3-Pinanone (9)	No	0.374	1.692	1.932
Borneol (19)	No	0.577	1.707	1.877
Borneol acetate (20)	No	0.526	1.904	1.875
Caffeic acid methyl ester (21)	No	−0.154	2.023	1.594
Camphor (23)	No	0.473	1.653	1.981
Caryophyllene oxide (25)	No	0.148	1.548	1.224
cis-Linalool oxide (26)	No	0.891	1.917	2.221
Dienestrol (29)	No	0.191	2.324	1.840
Hexanal (38)	No	0.833	1.762	1.922
Honokiol (39)	No	0.305	2.184	1.791
Isomagnolol (42)	Yes	0.724	2.025	1.929
Limonene (43)	No	0.777	1.880	2.336
Magnaldehyde D (47)	No	0.462	1.827	1.772
Magnolol (51)	No	0.468	1.976	1.851
Obovatol (62)	No	0.497	1.776	1.586
Phenol (67)	No	0.540	2.153	2.011
Randaiol (70)	No	0.391	2.383	1.457
α-Eudesmol (76)	No	0.131	1.680	1.231
α-Terpineol (78)	No	0.886	1.923	1.945
β-Eudesmol (79)	No	−0.220	1.727	1.304
γ-Eudesmol (80)	No	0.055	1.681	1.249
γ-Gurjunene epoxide (81)	No	0.347	1.647	1.428

**Table 3:** Molecular interaction between AD-related key targets and selected 23 compounds in *M. officinalis*.

Ligand	CID No.	Binding Energy (kcal/mol)			
		hBACE1 (6JSE)	hAChE (4EY7)	hQC (3PBB)	hGS3-β (1Q5K)
Khusilol (2)	556427	−6.939	−8.324	−8.288	−7.686
Ferulic acid methyl ester (6)	5357283	−6.146	−7.533	−6.973	−6.403
3-Pinanone (9)	11038	−5.956	−7.226	−5.301	−5.509
Borneol (19)	1201518	−5.303	−6.524	−4.472	−5.129
Borneol acetate (20)	93009	−5.862	−7.834	−5.122	−5.788
Caffeic acid methyl ester (21)	689075	−6.129	−7.489	−7.261	−6.393
Camphor (23)	2537	−5.220	−6.983	−4.877	−5.014
Caryophyllene oxide (25)	1742210	−7.275	−8.564	−5.876	−6.721
cis-Linalool oxide (26)	6428573	−5.685	−6.551	−5.801	−5.434
Dienestrol (29)	667476	−7.371	−9.494	−7.730	−8.395
Hexanal (38)	6184	−3.791	−4.576	−4.641	−3.992
Honokiol (39)	72303	−7.784	−9.442	−8.403	−8.303
Limonene (43)	22311	−5.887	−7.000	−6.650	−6.386
Magnaldehyde D (47)	5319189	−7.531	−9.390	−7.660	−7.079
Magnolol (51)	72300	−7.836	−9.825	−7.875	−7.284
Obovatol (62)	100771	−7.418	−9.827	−7.685	−7.675
Phenol (67)	996	−4.415	−5.250	−5.684	−4.501
Randaiol (70)	13337243	−7.733	−8.558	−7.251	−7.382
α-Eudesmol (76)	92762	−7.719	−9.856	−7.222	−7.884
α-Terpineol (78)	17100	−5.818	−7.086	−6.530	−6.173
β-Eudesmol (79)	91457	−7.829	−8.482	−7.496	−7.739
γ-Eudesmol (80)	6432005	−7.692	−9.668	−8.062	−7.473
γ-Gurjunene epoxide (81)	91750423	−6.940	−7.956	−6.669	−6.362
C6R ^1^	138857908	−10.400	-	-	-
E20 ^1^	1150567	-	−12.200	-	-
PDB ^1^	6539196	-	-	−6.300	--
TMU ^1^	68670561	-	-		−8.000

^1^ C6R [N-[3-[(4S,5R)-2-azanyl-4-methyl-5-phenyl-5,6-dihydro-1,3-thiazin-4-yl]-4-fluoranyl-phenyl]-5-(fluoranylmethoxy)pyrazine-2-carboxamide)], E20 [1-benzyl-4-[(5,6-dimethoxy-1-indanon-2-yl)methyl]piperidine)], PDB [1-(3,4-dimethoxyphenyl)-3-[3-(1H-imidazol-1-yl)propyl]thiourea), and TMU [N-(4-methoxylbenzyl)-N’-(5-nitro-1,3-thiazol-2-yl)urea] were native ligands of hBACE1, hAChE, hQC, and hGSK-3β, respectively.

**Table 4:** Inhibitory effect and kinetic parameters of the top molecule against hBACE1, hQC, and GSK-3β.

Compound	Target Marker	IC_50_ ^1^	Ki ^2^	Inhibition Mode ^3^
Honokiol	hBACE1 ^4^	20.64 ± 0.80 µM	27.3 µM	Non-competitive
	AChE ^4^	78.85 ± 1.23 µM	58.2 µM	Non-competitive
	hQC ^4^	90.74 ± 1.07 µM	49.4 µM	Competitive
	hGSK-3β ^4^	6.70 ± 0.03 µM	9.8 µM	Non-competitive

^1^ The 50% inhibition concentration (µM) of the enzyme inhibitor is expressed as the mean ± S.D. of triplicate experiments. ^2^ The Ki value (µM) represents the binding affinity between the inhibitor and the enzyme. ^3^ Indicates the type of inhibitor, as determined by the Dixon and Lineweaver–Burk plots. ^4^ Quercetin (IC_50_ = 19.08 ± 0.16 µM), galantamine (IC_50_ = 1.83 ± 0.06 µM), resveratrol (IC_50_ = 30.87 ± 0.35 µM), and rutin (IC_50_ = 10.75 ± 0.03 µM) were used as positive controls in the BACE1, AChE, hQC, and hGSK-3β assays, respectively.

## Data Availability

All data generated in this study are included in the article.

## Supplementary Materials

The following supporting information can be downloaded at: https://www.mdpi.com/article/10.3390/ijms251910507/s1.
