# Supplementary material for: Determination of Potential Lead Compound from Magnolia officinalis for Alzheimer’s Disease through Pharmacokinetic Prediction, Molecular Docking, Dynamic Simulation, and Experimental Validation
Source: Int J Mol Sci. 2024 Sep 29;25(19):10507. doi: 10.3390/ijms251910507 (PMC11477134; doi:10.3390/ijms251910507)
Supplement: Supplementary file 1 [file ijms-25-10507-s001.zip › ijms-3176659-supplementary.pdf]

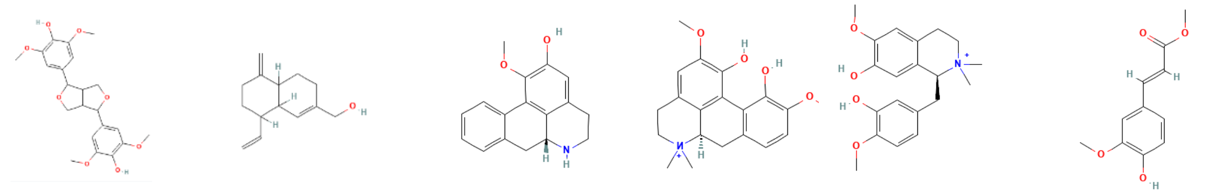

Syringaresinol (1) | Khusilol (2) | Asimilobine (3) | Magnoflorine (4) | Tembetarine (5) | Ferulic acid methyl ester (6)

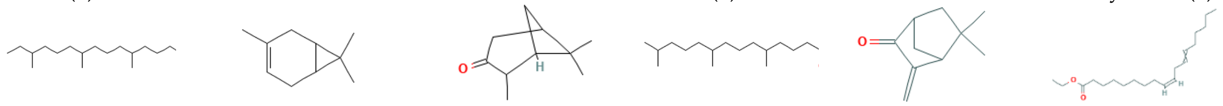

2,6,10,14-Tetramethyl-hexadecane (7) | 3-Carene (8) | 3-Pinanone (9) | 6,10,14-Trimethyl -2-penta decanone (10) | 6,6-Dimethyl-2-methylene-bicyclo[2.2.1]-heptan-3-one (11) | 9,12-Octadeca dienoic acid, ethyl ester (12)

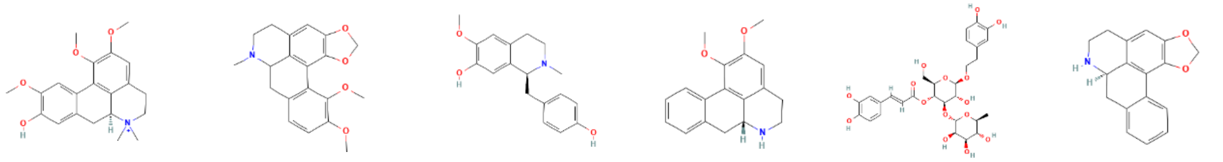

Xanthoplanine (13) | N-methyl bulbocapnine (14) | N-methyl coclaurine (15) | N-nornuciferine (16) | Acteoside (17) | Anonaine (18)

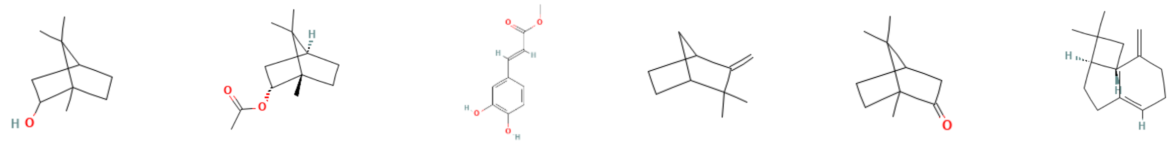

Borneol (19) | Borneol acetate (20) | Caffeic acid methyl ester (21) | Camphene (22) | Camphor (23) | Caryophyllene (24)

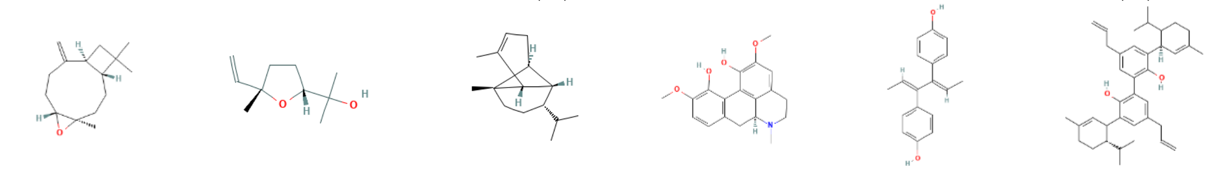

Caryophyllene oxide (25) | cis-Linalool oxide (26) | Copaene (27) | Corytuberine (28) | Dienestrol (29) | Dipiperityl magnolol (30)

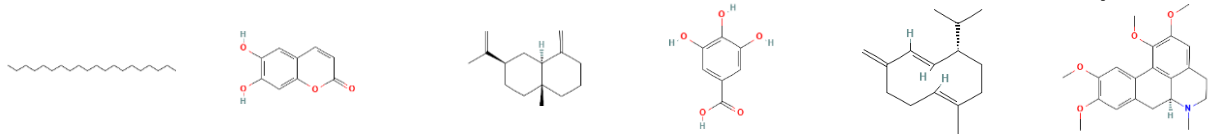

Eicosane (31) | Esculetin (32) | Eudesma-4(14),11-diene (33) | Gallic acid (34) | Germacrene D (35) | Glaucine (36)

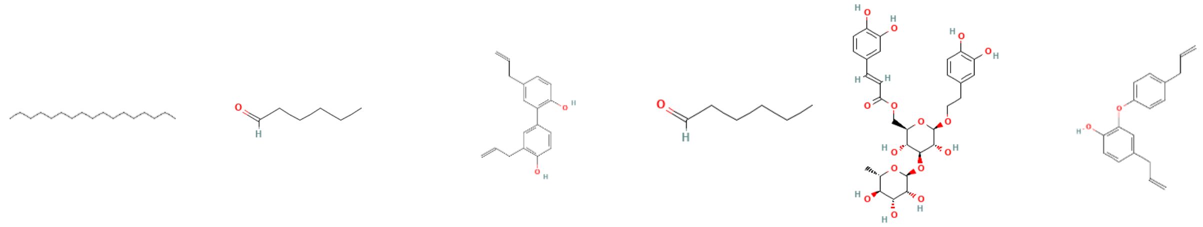

Heptadecane (37) | Hexanal (38) | Honokiol (39) | Humulene (40) | Isoacteoside (41) | Isomagnolol (42)

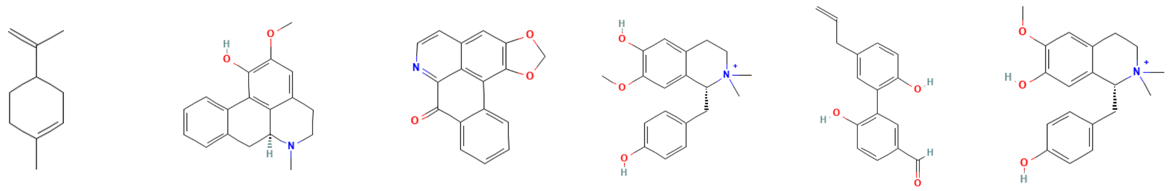

Limonene (43) | Lirinidine (44) | Liriodenine (45) | Lotusine (46) | Magnaldehyde D (47) | Magnocurarine (48)

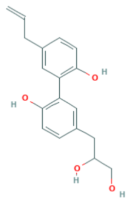
Magnolignan A (49)

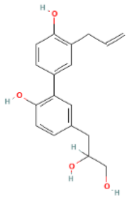
Magnolignan C (50)

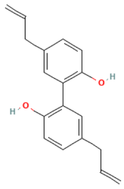
Magnolol (51)

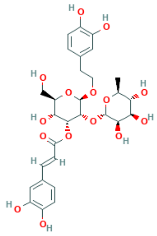
Magnoloside A (52)

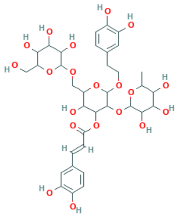
Magnoloside B (53)

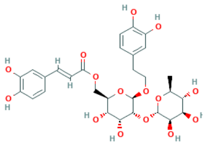
Magnoloside D (54)

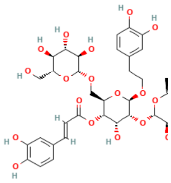
Magnoloside F (55)

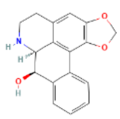
Michelalbine (56)

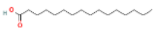
n-Hexadecanoic acid (57)

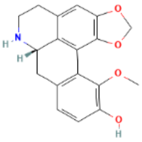
Nandigerine (58)

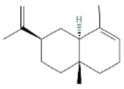
α -Selinene (59)

Nonadecane (60)

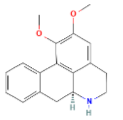
Nornuciferine (61)

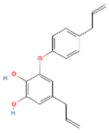
Obovatol (62)

Octadecane (63)

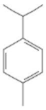
p-Cymene (64)

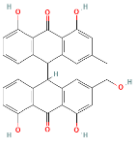
Palmidin B (65)

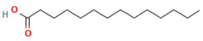
Pentadecanoic acid (66)

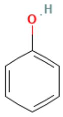
Phenol (67)

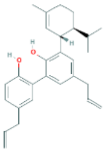
Piperityl Magnolol (68)

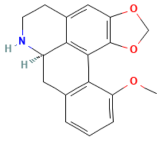
Puterine (69)

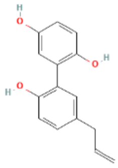
Randaiol (70)

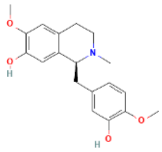
Reticuline (71)

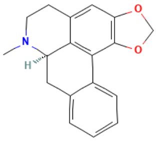
Roemerine (72)

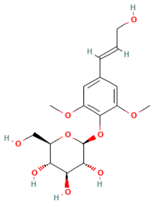
Syringin (73)

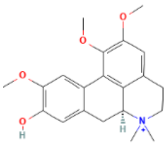
Xanthoplanine (74)

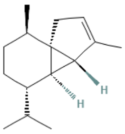
α-Cubebene (75)

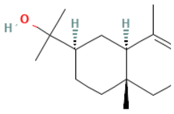
α-Eudesmol (76)

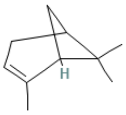
α-Pinene (77)

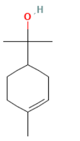
α-Terpineol (78)

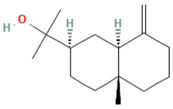
β -Eudesmol (79)

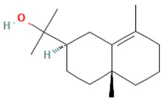
γ-Eudesmol (80)

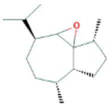
γ-Gurjunene epoxide (81)

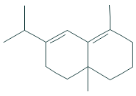
δ-Selinene (82)

Figure S1. Chemical structure of compounds in *Magnolia officinalis*
